# Re‐Determination of the Crystal Structure of MIL‐91(Al)

**DOI:** 10.1002/zaac.201600358

**Published:** 2016-12-16

**Authors:** Nele Hermer, Michael T. Wharmby, Norbert Stock

**Affiliations:** ^1^ Institute of Inorganic ChemistryChristian‐Albrechts‐UniversitätMax‐Eyth‐Straße 224118KielGermany; ^2^ Diamond Light Source Ltd.Harwell Science & Innovation CampusOX11 0DEDidcotOxfordshireUK

**Keywords:** Metal‐organic frameworks; Phosphonates; Porous materials; Rietveld refinement; Synchrotron powder X‐ray diffraction

## Abstract

The structure of one of the first permanently porous metal phosphonates, MIL‐91(Al) was re‐determined using high resolution synchrotron powder X‐ray diffraction data. The new model is in a lower symmetry space group, with no disordered ligands in the structure, whilst remaining otherwise consistent with the reported compound. New milder synthetic conditions were also developed.

## Introduction

In the intensely investigated field of porous materials, metal organic frameworks (MOFs) constitute a class of materials with exceptional properties and a remarkable structural diversity.^1^ Metal phosphonates are an important subclass of MOFs and have received much attention due to their potential in fields of application such as proton conductivity,^2^ ion exchange,^3^ radioactive waste treatment,^4^ catalysis^5^ and molecular adsorption/separation.^6^ For these applications, both high thermal and chemical stability are required. Metal carboxylate based MOFs are often insufficiently stable under the application conditions;^7^ by contrast, phosphonate linkers present advantages in terms of the stability of the framework compounds they form. This is due to the higher charge of the coordinating group, the higher number of atoms involved in bonding and the tendency to form more than one oxygen‐metal bond per oxygen atom.^8^

Storage and separation of CO_2_ is of great current interest due to increasing awareness of global warming,^9^ and it is hoped that MOFs might play a role in addressing this problem. MIL‐91 (MIL – Matériaux de l′Institut Lavoisier) is a narrow pore metal phosphonate framework formed from *N*,*N*′‐piperazinebis(methylenephosphonic acid) (H_4_ **L**; H_2_O_3_PCH_2_–C_4_H_8_N_2_–CH_2_PO_3_H_2_) and either Al^3+^ or Ti^4+^ as the framework forming cation [*M*OH*_n_*(H_2_ **L**)]**·** *x*H_2_O (*M* = Al^3+^, *n* = 1, *x* ≈ 3 or *M* = Ti^4+^, *n* = 0, *x* ≈ 4.5).^10^ The Al compound was recently investigated by computational and experimental methods for its adsorption properties in flue‐gas treatment.^11^ MIL‐91(Al) demonstrates significant uptake of CO_2_ at low pressure as well as high selectivity for CO_2_, and is also thought to exhibit some structural flexibility during the CO_2_ adsorption process.

The present study reports a redetermination of the structure of the MIL‐91(Al), which removes the disorder present in the reported structure and may allow a deeper understanding of the nature of this flexibility.

## Results and Discussion

Initial syntheses of MIL‐91(Al) followed the reported synthetic procedure.^10^ Through a high‐throughput investigation these synthetic conditions were further optimized, leading to a substantially reduced reaction time (64 h instead of 130 h) at 190 °C. The high‐throughput reactions also led to an optimized reaction stoichiometry of 0.5:1 (AlCl_3_:H_4_ **L**). Under these conditions, the addition of base was not necessary. As a final optimization, the most crystalline samples were obtained when a heating and cooling ramp, each of 12 h, was used at the start and end of the reaction.

**Figure 1:**
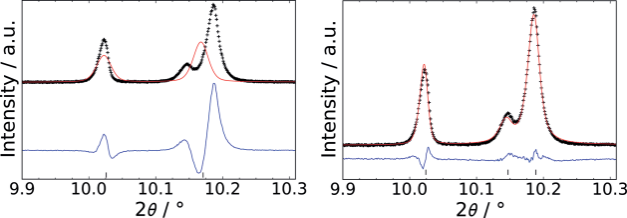
Le Bail fits of the peaks in the range 9.9–10.3° 2*θ* for MIL‐91(Al) cell in *C2/m* (left) and *P* 1 (right). In *P* 1 structure two additional peaks are present due to the inequivalence of the 112/11 2 and the 122/122 reflections (*λ* = 0.825781 Å).

From all reactions, MIL‐91(Al) was obtained as a microcrystalline powder consisting of needle‐shaped crystals. To re‐determine the structure of the compound, synchrotron X‐ray powder diffraction data were collected at beamline I11 (Diamond Light Source, Oxon., UK), using the MAC detector to give the highest possible angular resolution.^12^ All analyses of these data were performed with the program TOPAS.^13^ Initial analyses used the published monoclinic unit cell parameters for the compound [*a* = 18.959(9), *b* = 6.915(3), *c* = 11.256(6) Å, *β* = 90.54(2)°, *V* = 1467.8(13) Å^3^; *C2/m*].^10^ However, a Le Bail fit using this cell gave a poor fit to the data (*R*_wp_ = 24.8 %; Supporting Information S7). The data were therefore re‐indexed in a unit cell with triclinic symmetry and approximately half the volume of the reported structure (*V* = 737.705(4) Å^3^, *R*_wp_ = 10.1 %; Table 1, Supporting Information S7). Looking closely at reflections at 10.15°, 13.2°, and 14.25–15.25° 2*θ* (Figure 1; Supporting Information S7), it is clear that numerous peaks, which appeared as single reflections in low resolution diffraction data, are actually groups of closely spaced peaks that can only be accounted for using the new, lower symmetry, triclinic model. The origin of this “splitting” is the *α* and *β* angles having values very close to 90°, which leads to near‐pseudosymmetry. The original structure of MIL‐91 was obtained from a very small single crystal.^10^ Given the acicular habit of MIL‐91(Al), it is conceivable that this was actually a twinned crystal and thus in combination with the near‐pseudosymmetry of the unit cell the structure determination in the monoclinic space group would be almost inevitable.

**Table 1:** Summary of crystallographic information for MIL‐91(Al)

	MIL‐91(Al)
Formula	[Al(OH)(C_6_H_14_N_2_P_2_O_6_)]**·**3H_2_O
*a* /Å	6.921408(17)
*b* /Å	10.05342(3)
*c* / Å	11.27829(4)
*α* /°	89.6820(4)
*β* /°	89.9141(3)
γ /°	70.0966(3)
Volume /Å^3^	737.899(4)
Crystal system	triclinic
Space group	*P* 1
Independent atoms	23
*R*_wp_, *R*_Bragg_, χ^2^	9.22, 4.04, 4.599

**Figure 2:**
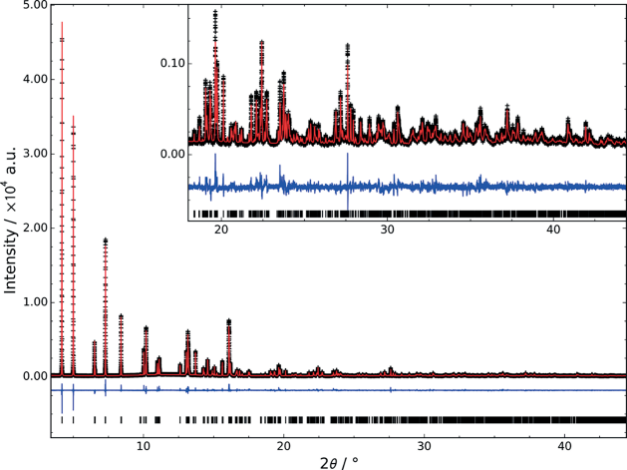
Rietveld difference plot for the refinement of MIL‐91(Al). Observed data (black), calculated plot (red), difference plot (blue), and expected peak positions indicated with black bars (*λ* = 0.825781 Å).

A new model for the MIL‐91 structure was developed using the new triclinic unit cell, with atomic positions derived from the literature structure.^10^ The program Materials Studio^14^ was used to reduce the symmetry of the structure to *P*1 and to remove the disorder of the linker molecules. Cell parameters and symmetry of this hypothetical structure were revised with PLATON^15^ to space group *P* 1 and were found to be in excellent agreement with the indexed values. A Rietveld refinement was then performed, applying chemically sensible restraints to bond lengths. Water molecules were located within the channels of the network by Fourier difference mapping, with sites O103 and O104 being disordered. The final cycles of refinement gave a good final fit to the data (Table 1; Figure 2).

**Figure 3:**
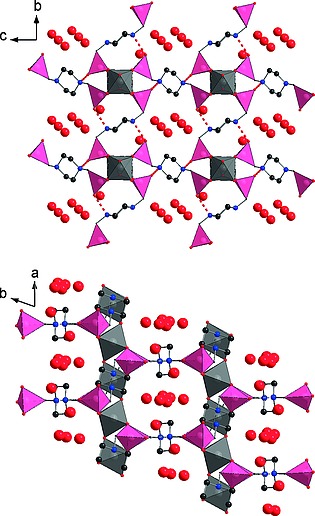
Structure of MIL‐91(Al), viewed along the *a* direction (top; parallel to the chains) and along the *c*‐direction (bottom). Dashed red bonds indicated P–O**···**H–N hydrogen bonding interactions. O in red, N in blue, C in black, AlO_6_ octahedra in grey, and CPO_3_ tetrahedra in pink.

Structurally both the *C*2/*m* and *P* 1 models of MIL‐91(Al) are very similar, although the structure is rotated with respect to the unit cell axes (Supporting Information, Figure S8.2). The asymmetric unit consists of two Al^3+^ cations, one in the center of the cell and one in the center of the *bc* faces of the cell, and two symmetry independent half H_2_ **L** ^2–^ ligands. Each Al^3+^ cation is octahedrally coordinated by four oxygen atoms from four different phosphonate groups and two hydroxide ions. Al–OH–Al chains are formed through the corner‐sharing OH groups, which lie parallel to the *a* direction (Figure 3); this is in contrast to the reported structure (Supporting Information S8), where chains are parallel to the *b* direction. Neighboring Al^3+^ cations are also connected by CPO_3_ tetrahedra, which coordinate in bridging mode, with one pendant O atom. The two independent H_2_ **L** ^2–^ ligands link the chains in the *b* and *c* directions respectively, forming a unidirectional channel structure, with a diameter of ca. 3.5 Å. The two protons remaining on the linker engage in hydrogen bonding interactions, indicated by short interatomic distances between the piperazinyl N atoms and pendant O atom of the CPO_3_ groups (N11**···**H**···**O23–P21: 2.564(9) Å; N21**···**H**···**O13–P11: 2.597(7) Å, indicated in Figure 3). It is unclear whether protons are bonded to the N or O atom; nevertheless this interaction is thought to stabilize the framework. Water molecules are distributed over four sites within the channels of the structure and form a hydrogen bonding network, interacting with each other (O**···**O: 2.43(3)–2.877(8) Å) and also with the O atoms of the CPO_3_ groups (O**···**O: 2.846(8)–2.864(10) Å).

To confirm the synthesized compound has the same properties as the literature MIL‐91(Al) and to rule out the possibility of impurities, thermogravimetric analysis and elemental analysis measurements were performed. Thermogravimetric measurements (Supporting Information S3) indicate the loss of three water molecules per formula unit (obsd. 15.0 wt.‐%; calcd. 14.6 wt.‐%), in agreement with both the reported composition and that derived from the model from the powder X‐ray diffraction data. As previously observed, after the loss of water molecules, MIL‐91(Al) shows a step in the TGA (obsd. 5.3 wt.‐%; calcd. 4.8 wt.‐%) ascribed to the dehydroxylation of the framework. Elemental analysis results are also consistent with the literature MIL‐91(Al) (Supporting Information S1), confirming the high purity of the sample. These results allow us to rule out an impurity as the origin for the peak “splitting” observed.

**Figure 4:**
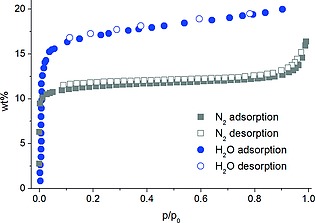
Grey symbols: N_2_ sorption isotherm of MIL‐91(Al) (measured at 77 K), blue symbols: water sorption isotherm of MIL‐91(Al) (measured at 298 K), activation under vacuum (0.1 mbar at 140 °C for 12 h).

To evaluate the porosity of the samples prepared by our new synthetic method, N_2_ sorption isotherms at 77 K were recorded (Figure 4). MIL‐91(Al) was activated at 140 °C for 12 h under vacuum. A specific surface area of a_BET_ = 356 m^2^ g^–1^ and a micropore volume of *V*_mic_ = 0.146 cm^3^ g^–1^ were determined, which are slightly better than the most recently reported values (a_BET_ = 310 m^2^ **·**g^–1^, *V*_mic_ = 0.12 cm^3^ **·**g^–1^).^11^ The crystallographic micropore volume of the triclinic model (*V*_mic_ = 0.150 cm^3^ **·**g^–1^; determined using PLATON) also agrees well with the experimentally determined value. Additionally H_2_O sorption measurements were performed at 298 K, with MIL‐91(Al) shown to be porous to water with an uptake of 20 wt.‐% at 100 kPa (Figure 4). The isotherm indicates a strongly hydrophilic behavior as the uptake occurs in the range 0 < *p/p*_0_ < 0.08. As part of both sorption experiments, the effect of activation on the compound was evaluated by powder X‐ray diffraction (Supporting Information S9, Figure S9.2). No substantial changes were observed in the diffraction patterns, indicating little or no degradation occurs during the measurements.

Through the use of high resolution synchrotron powder X‐ray diffraction data, the MIL‐91(Al) structure was successfully re‐determined in the triclinic space group *P* 1. The use of high‐resolution synchrotron data was crucial to this study, as it revealed a near‐pseudosymmetry of the *P* 1 unit cell, which led to the previous structure solution in the monoclinic space group *C*2/*m*. By reducing the symmetry, the disorder present in the literature structure could be removed. The triclinic structural model was confirmed by a Rietveld refinement against synchrotron data. Thermogravimetric and elemental analysis studies confirmed that there are no impurities present in the sample. As part of this work, an optimized synthetic procedure for MIL‐91(Al) was developed. N_2_ adsorption experiments confirmed the porosity of the compound and indicate that the MIL‐91(Al) synthesized by the new method has a capacity for N_2_ consistent with the literature compound.^11^ Additionally, the water sorption properties of MIL‐91(Al) were measured for the first time, indicating it to be highly hydrophilic.

**Supporting Information** (see footnote on the first page of this article): Characterisation methods, synthesis, thermogravimetric analysis, IR spectroscopy, crystallographic methodology, summary of crystallographic results, comparison of Le Bail Fits of reported *C*2/*m* and new *P* 1 MIL‐91(Al) structures and N_2_ isotherm of MIL‐91(Al).

## Supporting information

Supporting information for this article is available on the WWW under https://doi.org/10.1002/zaac.201600358 or from the author.

Supporting Information
