# Supplementary material for: Re‐Determination of the Crystal Structure of MIL‐91(Al)
Source: Z Anorg Allg Chem. 2016 Dec 16;643(2):137–40. doi: 10.1002/zaac.201600358 (PMC5324569; doi:10.1002/zaac.201600358)
Supplement: Supplementary file 1 — Supporting Information [file ZAAC-643-137-s001.pdf]

Z. Anorg. Allg. Chem. **2016** · ISSN 0044–2313

SUPPORTING INFORMATION

Title: Re-Determination of the Crystal Structure of MIL-91(Al)

Author(s): N. Hermer, M. T. Wharmby,* N. Stock*

Ref. No.: z201600358

S1 Methods

S2 Synthesis

S3 Thermogravimetric Analysis

S4 Infrared Spectrum

S5 Crystallographic Methodology

S6 Summary of Crystallographic Results

S7 Comparison of Le Bail Fits of Reported *C2/m* and New *P-1* MIL-91(Al) Structures

S8 Comparison of the Crystal Structures of Reported *C2/m* and *P-1* MIL-91(Al)

S9 Porosity of MIL-91(Al) & Effect of Activation on the Structure

S10 References

S1 Methods

N, N′-piperazinebis(methylenephosphonic acid) was synthesised according to literature method.[1] All other reagents and solvents were commercially available and used without further purification. Elemental analysis (C H N S) was performed on a EuroEA3000, using Sulfanilamide as the reference. Thermogravimetric measurements were performed under a stream of air (75 mL min^{-1}) using a Netsch STA-409CD instrument running, with data collected over the range 25 to 900°C and a heating rate of 4°C min^{-1}. For sorption experiments, 20-40 mg MIL-91(Al) were activated at 140°C for 12 hrs under reduced pressure (10^{-2} kPa). N_2 and H_2O sorption measurements were carried out using a BELSORP-max (BEL Japan Inc.). The surface area was determined with the BET method,[2] for which the pressure range for application of the BET method was determined by a Rouquerol plot (end point at $p/p_0 = 0.0399$).[3] Pore volume was determined at $p/p_0 = 0.5$. The IR spectra were recorded on a Bruker ALPHA-P FTIR spectrometer in the spectral range 4000–400 cm^{-1}.

S2 Synthesis

N, N′-piperazinebismethylenephosphonic acid (40 mg, 0.15 mmol) and $AlCl_3 \cdot 6H_2O$ (17.6 mg, 0.073 mmol) were placed in Teflon® lined autoclave (V_{max}=2 ml) with deionised water (1 ml) to give a reaction concentration of 0.223 mol dm^{-3}. The liner was then inserted into one of our in-house developed 24 reaction high-throughput multiclaves.[4] The reactor was slowly heated to 190°C over 12 hrs and the temperature was kept constant for 64 h, before the reactor was cooled to room temperature, again over 12 hours. The precipitate was filtered and washed with water. The final product was a colourless microcrystalline powder, consisting of clumps of needle shaped crystallites (14.5 mg, 0.039 mmol; yield 53 %, based on $AlCl_3 \cdot 6H_2O$).

Elemental analysis gave C 18.85 % H 6.34 % N 7.12 %; expected for $[Al(OH)(C_6H_{14}N_2P_2O_6)] \cdot 3H_2O$: C 18.56 % H 5.97 % N 7.21 %.

S3 Thermogravimetric Analysis

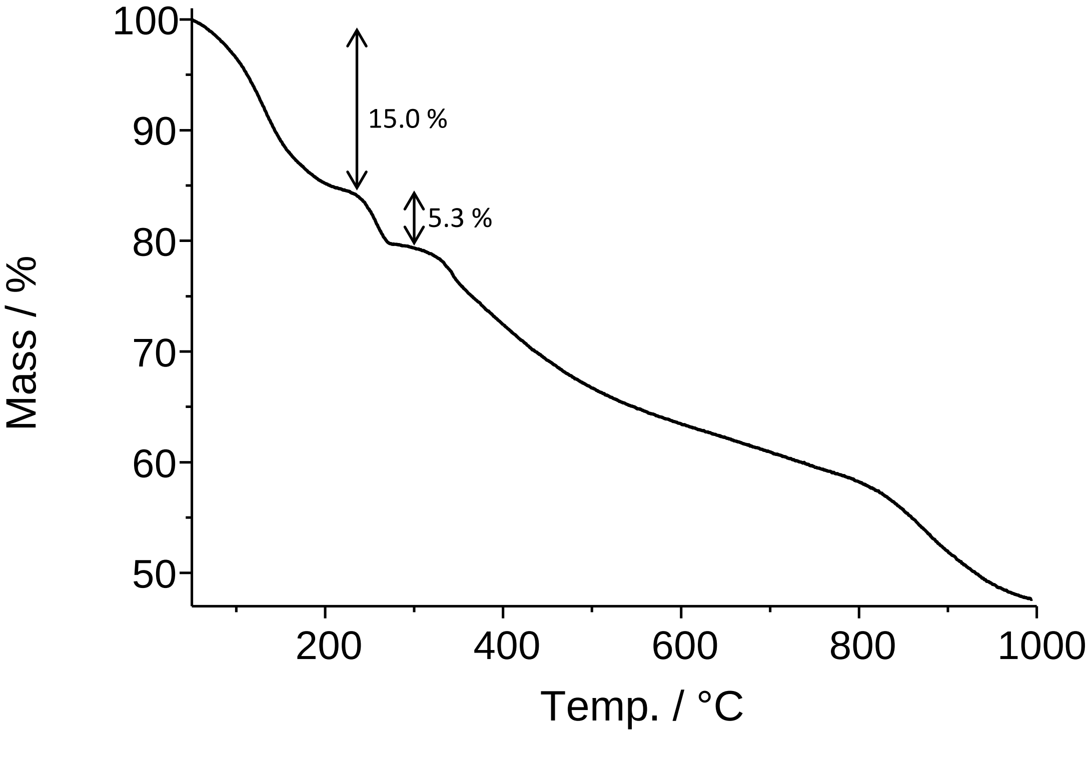

Weight loss from 25 to 210°C corresponds to the loss of approximately three molecules of water per formula unit (observed 15.0 wt.%; expected 14.6 wt.%). From 210 °C to 280 °C is attributed to the dehydroxylation of the framework (observed -5.3 wt. %; expected -4.9 wt. %). Above 350°C the organic portion of the framework is lost (observed 32.6 wt.%; expected 30.3 wt.%, for $C_6H_{12}N_2$). Final product is thought to be a complex X-ray amorphous mixture of aluminium ortho- and metaphosphate ($AlPO_4$ and AlP_3O_9) with composition $Al_2P_4O_{13}$.

S4 Infrared Spectrum

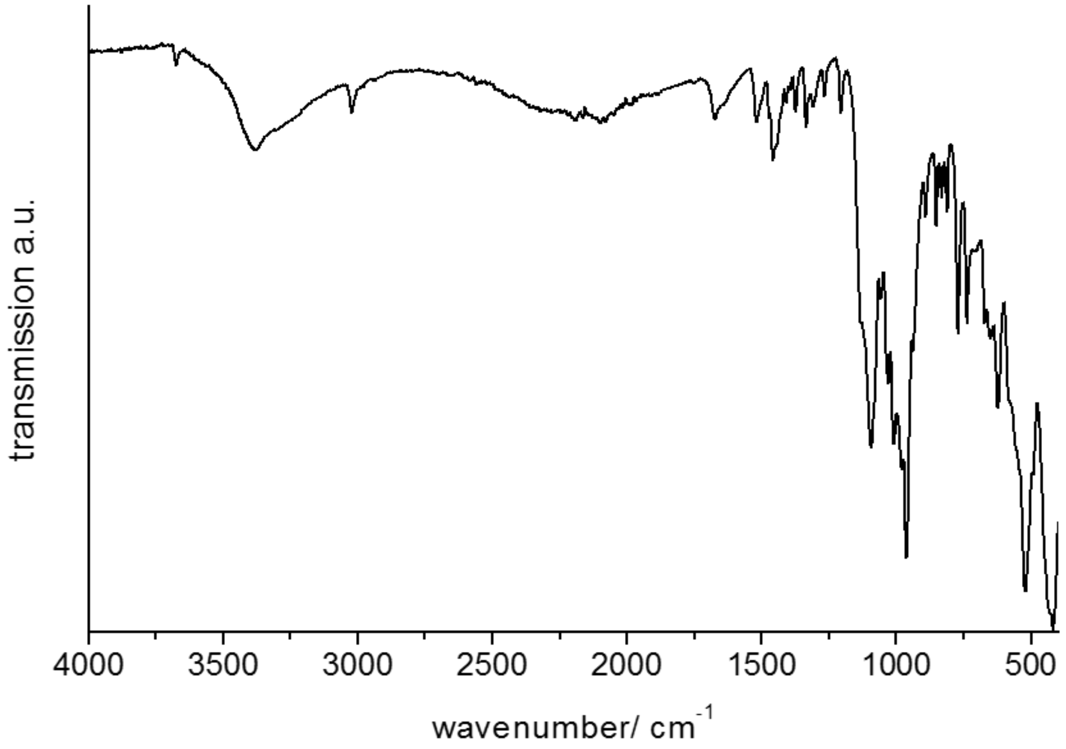

Figure S1. IR spectrum of MIL-91(Al).

IR ṽ (cm^{-1}) = 3675 (w), 3375 (b), 3019 (w), 1674 (b), 1521 (w), 1473 (w), 1455 (w), 1442 (w), 1407 (w), 1394 (w), 1374 (w), 1333 (w), 1305 (w), 1266 (w) 1204 (w), 1133 (m), 1095 (s), 1057 (m), 1028 (m), 1010 (m), 977 (m), 961 (s), 935 (m), 889 (w), 850 (w), 832 (w), 810 (w), 770 (m), 736 (m), 673 (w), 648 (w), 620 (m), 581 (w), 558 (w), 548 (w), 519 (s), 490 (m), 431 (s), 418 (s).

N.B. The sharp band at 3675 cm^{-1} can be assigned to the O-H stretch of the corner-shared hydroxyl groups.

S5 Crystallographic Methodology

A sample of MIL-91(Al) lightly ground before being loaded into a 0.7 mm borosilicate glass capillary tube. This was then mounted on the diffractometer at beamline I11, Diamond Light Source (Oxon., UK).[5] Diffraction data were collected in a Debye-Scherrer geometry using the Multi-Crystal Analyser (MAC) detector with monochromatic synchrotron X-ray radiation (λ = 0.825781Å; calibrant: NIST SRM 640c). By collecting data with the MAC it was possible to measure a wide range of data (0-150° 2θ) with a very high resolution (step size 0.001°). Data were collected for 30 mins on the same portion of the capillary; no appreciable beam-damage was observed.

For analysis, data in the range 3.4-44.4° 2θ were selected, as this range of data showed relatively well defined peaks with identifiable profiles. All data analysis was performed using the program TOPAS-Academic v5.[6] Peaks were very carefully selected and indexing yielded a triclinic cell with a volume approximately half that of the reported monoclinic cell (Table S5.1). A Le Bail fit of this cell was then performed, with the data fitted to a Pseudo-Voigt peak shape.[7] During the Le Bail fitting, it was found that some of the peaks show asymmetric broadening; this was fitted using the Stephens model implemented within TOPAS.[8] The final Le Bail fit gave an R_{wp} = 10.06 % (Table S5.1). Using the same peak profile functions and background, but with the reported monoclinic cell gave a significantly worse fit (R_{wp} = 24.81 %) (see Section S7 for detailed comparison).[9]

Table S5.1. Comparison of the literature cell parameters of Serre *et al.* with the newly indexed cell parameters, after Le Bail fitting.

	Literature Structure[9]	This work
Crystal System	Monoclinic	Triclinic
Space group	*C2/m*	*P-1*
a / Å	18.859(9)	6.920912(18)
b / Å	6.915(3)	10.07905(3)
c / Å	11.256(6)	11.27706(4)
α / °	-	90.2622(3)
β / °	90.54(2)	89.9121(3)
γ / °	-	69.6855(3)
Volume / Å^3	1467.8(13)	737.705(4)

A model for MIL-91(Al) with a triclinic symmetry was developed from the reported structure, using Materials Studio and PLATON.[10,11] The symmetry of the structure was first lowered and the disorder removed. Cell parameters and symmetry were then revised, using PLATON, and found to be in good

agreement with the cell parameters derived from the Le Bail fit. Finally the structure was geometry optimised.

The model was refined by the Rietveld method, using the peak function, background and cell derived from the Le Bail fit as starting model. Restraints were applied to all bonding distances and to some non-bonding distances (Table S5.2). Once the structure of the framework was well refined, water molecules (present as O atoms in the model) were deleted and after a couple of cycles of refinement, located by Fourier difference maps. It was found that a model including two disordered water molecules (O103 & O104), with a total occupancy of one O atom, gave an improved fit compared to a single fully occupied O atom.

Table S5.2. List of restraints applied to the model during Rietveld refinement.

Bonding Distances		Non-Bonding Distances	
**Distance**	**Restraint ± ESD / Å**	**Distance**	**Restraint ± ESD / Å**
Al-O	1.90±0.005	O···O (P td.)	2.53±0.01
P-O	1.53±0.001	O···C (P td.)	2.78±0.01
P-C	1.85±0.001	N···N (x-ring)	2.88±0.005
C-N (ex. ring)	1.52±0.001	C···C (x-ring)	2.88±0.005
C-N (int. ring)	1.50±0.001	C···N (2nd neighbour)	2.45±0.005
C-C	1.50±0.001	C···C (2nd neighbour)	2.45±0.005

During the Rietveld refinement, the peak shape model was changed to use the full axial divergence model (rather than simple axial) to model the asymmetry of the first two peaks.[12,13] In the final cycles of refinement, the isotropic ADPs (represented as beq in the CIF) were also refined: framework ADPs were constrained to refine as one group and water molecule ADPs as a second. The final refinement gave a good fit to the data (R_{wp} = 9.22 %; χ^2 = 4.599 – Fig. S5.1).

The slightly high R_{wp} reflects difficulties encountered in fitting the peak shape. Whilst the Stephens model fits isotropic strain within a crystal, the differences in the peak shape may indicate anisotropic strain. It was decided that as most peaks (excluding the (001) and the (010)) were well fitted and the χ^2 is good, further modelling would be deleterious to the refinement as it would introduce additional free parameters that could mask structural data.

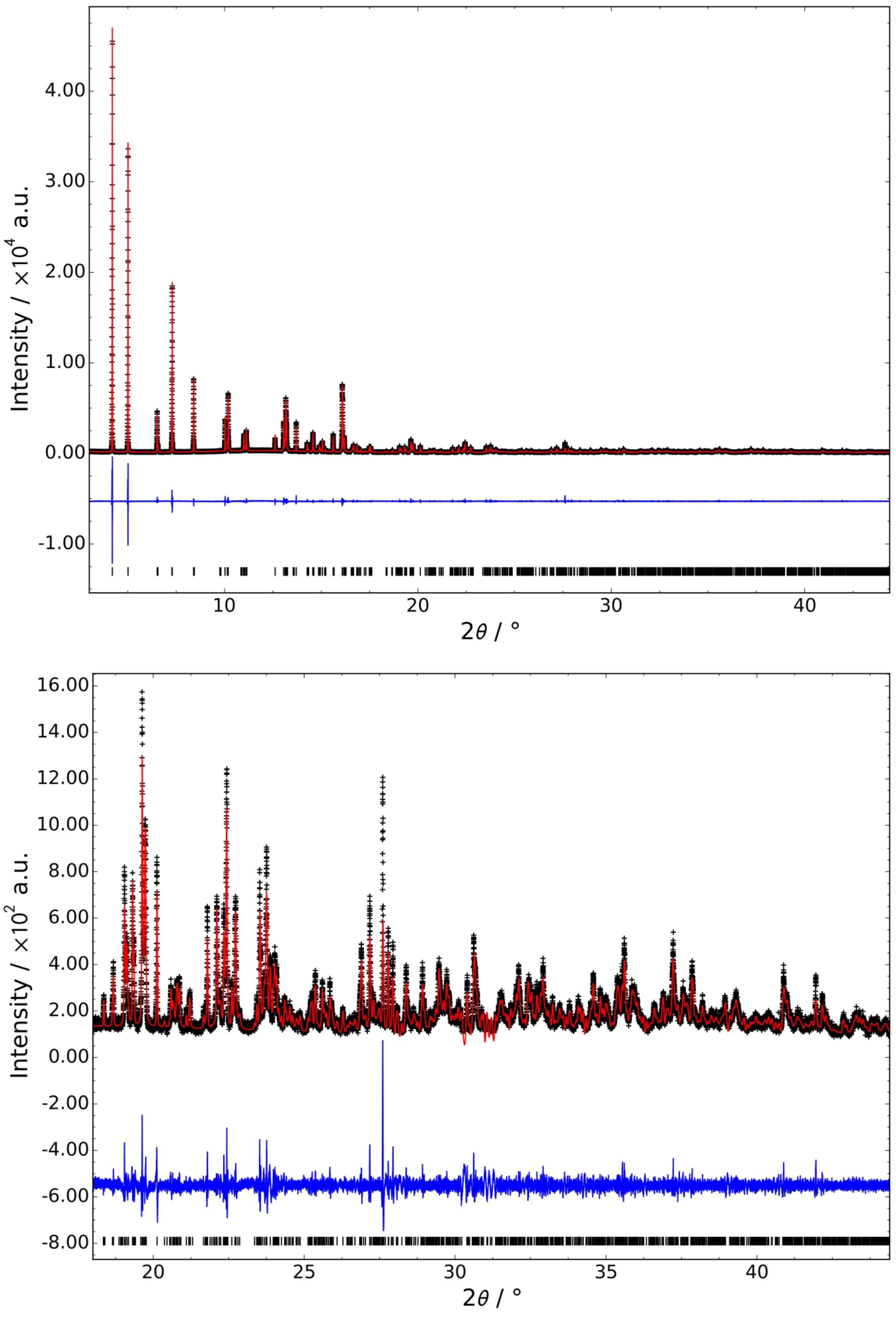

Fig. S5.1. Final Rietveld plots for the refinement of MIL-91(Al). (R_{wp} = 9.22 %; χ^2 = 4.599).

S6 Summary of Crystallographic Results

Table S6. Crystallographic data for for MIL-91(Al).

Structural formula	$[Al(OH)(H_2\mathbf{L})]\cdot 3H_2O^{\#}$
Moiety formula	$C_6\ H_{15}\ Al\ N_2\ O_7\ P_2,\ 3(H_2O)$
Sum formula	$C_6\ H_{21}\ Al\ N_2\ O_{10}\ P_2$
Formula weight	370.17
Calculated Density / g cm^{-3}	1.666
Space Group	*P*-1
a / Å	6.921408(17)
b / Å	10.05342(3)
c / Å	11.27829(4)
α / °	89.6820(4)
β / °	89.9141(3)
γ / °	70.0966(3)
V /Å^3	737.899(4)
Z	2
Diffractometer	I11 / Diamond Light Source
Temperature	298
Wavelength / Å	0.825781
No. Reflections	1221
No. Atoms	23
No. Restraints	52
R_p / %	7.02
R_{wp} / %	9.22
χ^2	4.599
R_{Bragg} / %	4.04

$^{\#}$ H_4L= N,N'-piperazinebis(methylenephosphonic acid) – $C_6H_{16}N_2O_6P_2$

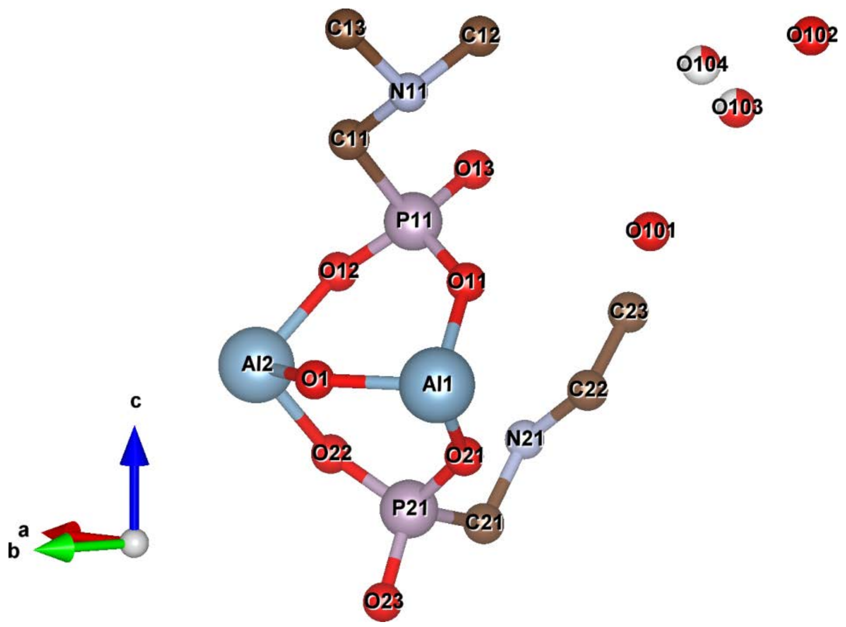

Fig. S6.1. Asymmetric unit of MIL-91(Al); note the two symmetry independent half N,N'-piperazinebis(methylenephosphonate) linkers. Al atoms are located on special positions within the cell. Occupancy of the disordered O104 and O103 sites is indicated by filling of sphere. (Key: dark blue – Al; red – O; pink – P; pale blue – N; brown – C.

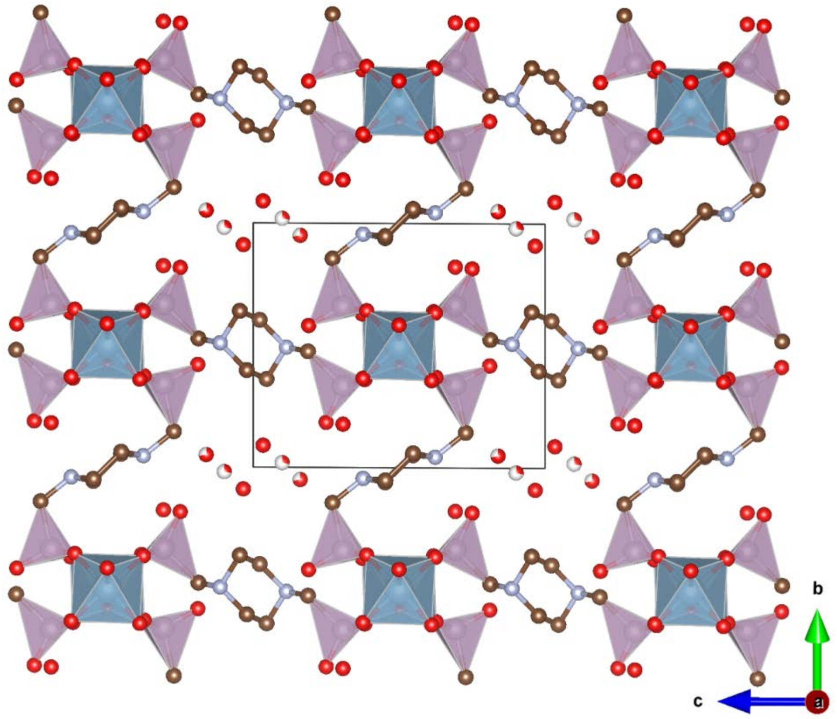

Fig. S6.2. View of MIL-91(Al) parallel to the *a*-axis, showing the water molecules in the channels. (Key – as Fig. S6.1; black box indicates the unit cell).

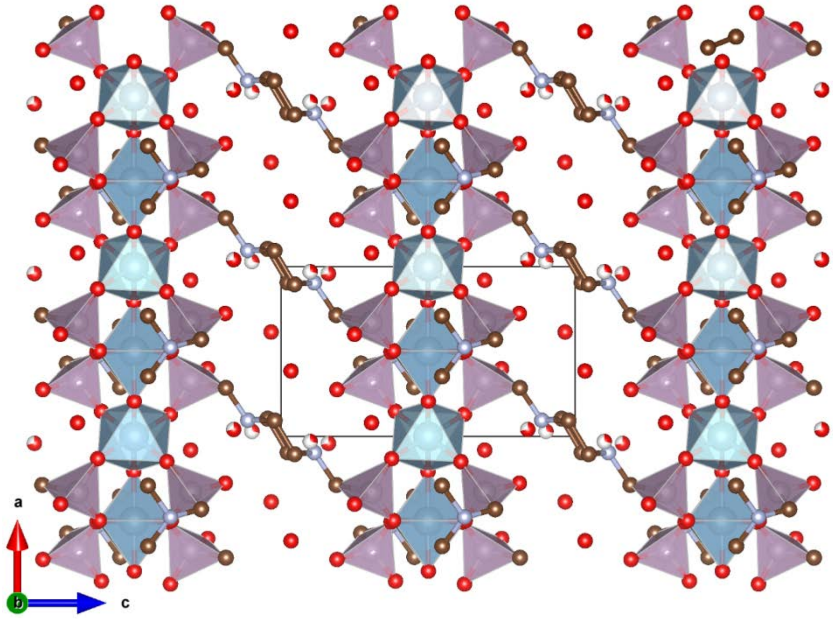

Fig. S6.3. View of MIL-91(Al) parallel to the *b*-axis, showing the structure of the chains and the connectivity between them. (Key – as Fig. S6.1; black box indicates the unit cell).

S7 Comparison of Le Bail Fits of Reported *C2/m* and New *P*$\bar{1}$, MIL-91(Al) Structures

**Reported Structure** *C2/m*	**Structure From This Work** ***P*-1**

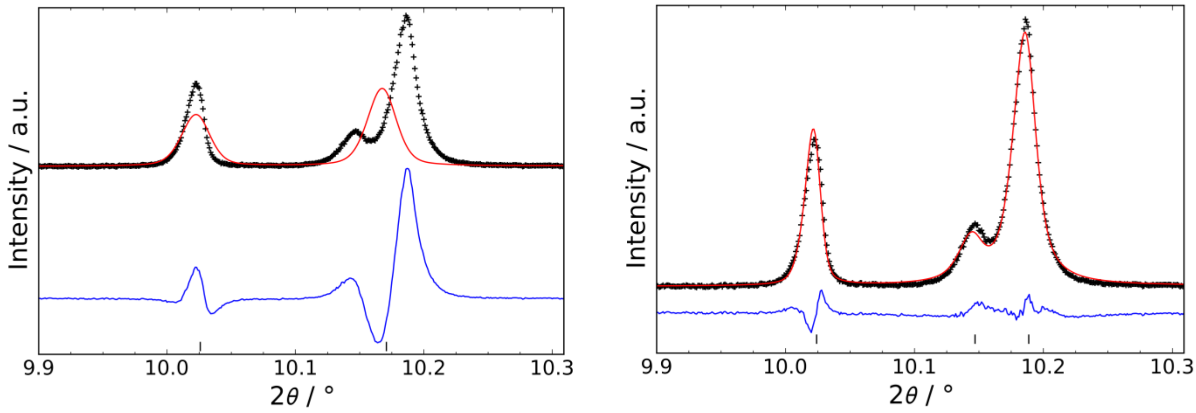

Range: 9.9-10.3°

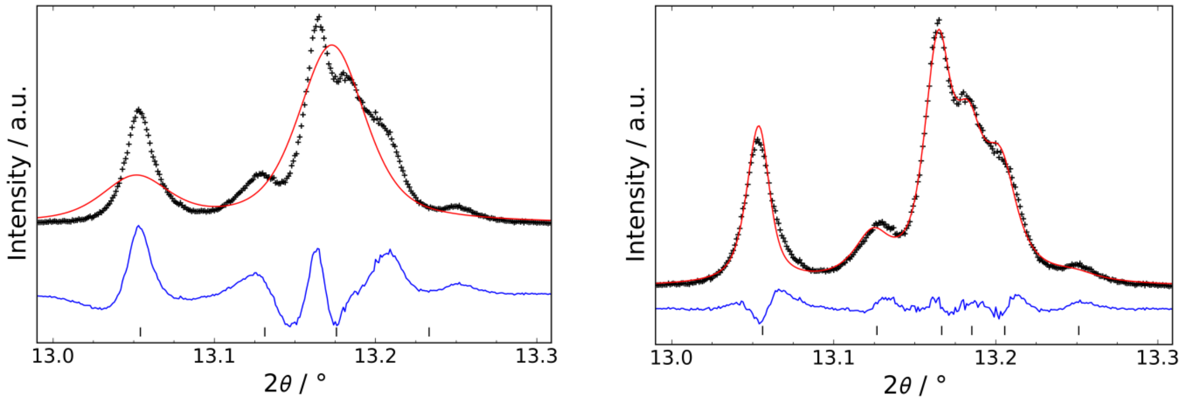

Range: 13-13.3°

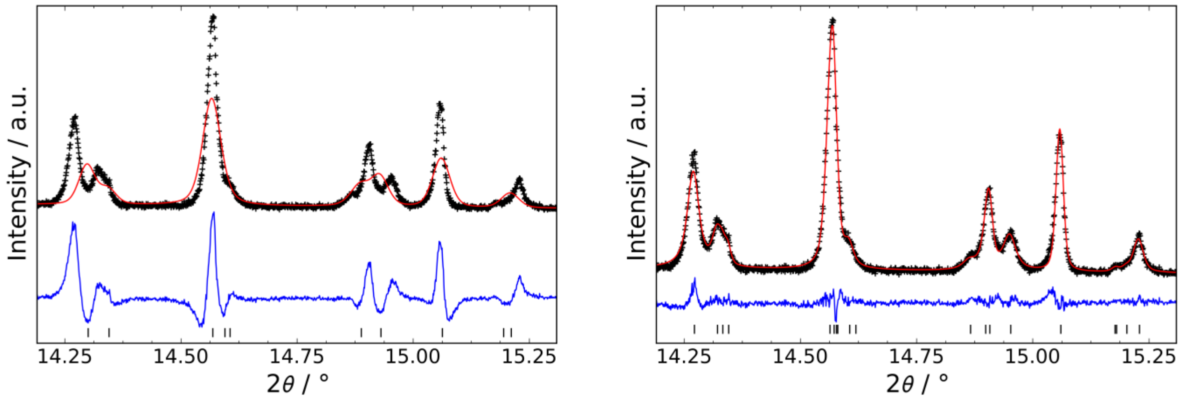

Range: 14.2-15.3°

λ = 0.825781 Å

The plots above illustrate the differences in the Le Bail fits over crucial regions of the diffraction data. Peak profile (Pseudo-Voigt peak shape + Stephens anisotropic broadening[8]) and background parameters were first refined for the triclinic cell to obtain good starting values. These were then

used as a starting point for a refinement which included the unit cell and zero point of a) the monoclinic cell (results shown left) and b) the triclinic cell (results shown right) (N.B. for the monoclinic refinement, the Stephens model used was for monoclinic crystal systems). In the final cycles of refinement, all parameters were allowed to refine freely.

The Le Bail fit of the monoclinic cell gave a poor fit to the data (R_{wp} = 24.81 %) whilst the triclinic cell gave a much more acceptable fit (R_{wp} = 10.06 %). From the plots above, it is clear that there are indeed more peaks that can be fitted with a monoclinic model and therefore the triclinic unit cell which we propose in this work is the best candidate.

S8 Comparison of the Crystal Structures of Reported *C2/m* and New *P1,* ¯ MIL-91(Al)

C2/m	***P-1***
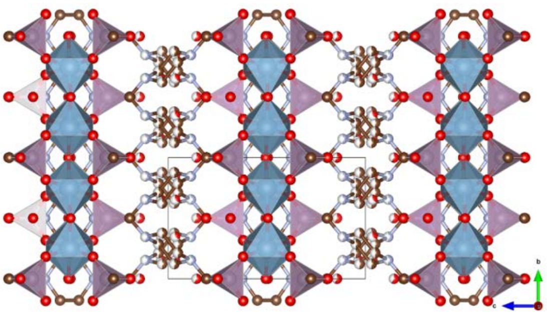 View parallel to *a*-axis	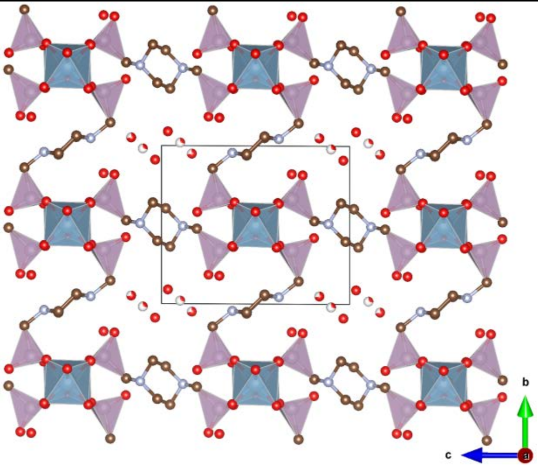 View parallel to *a*-axis
C2/m	***P-1***
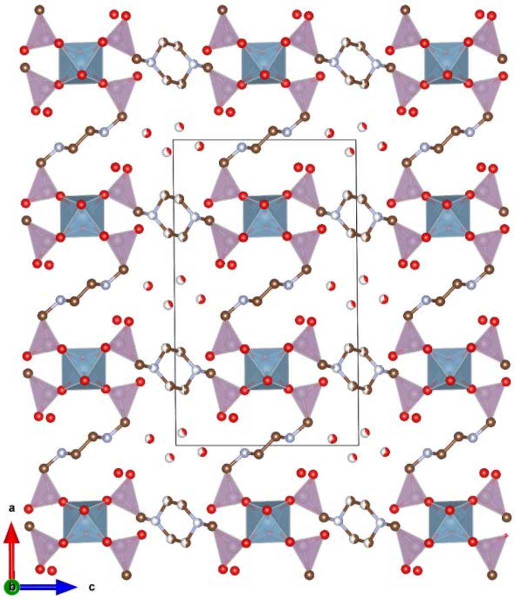 View parallel to *b*-axis	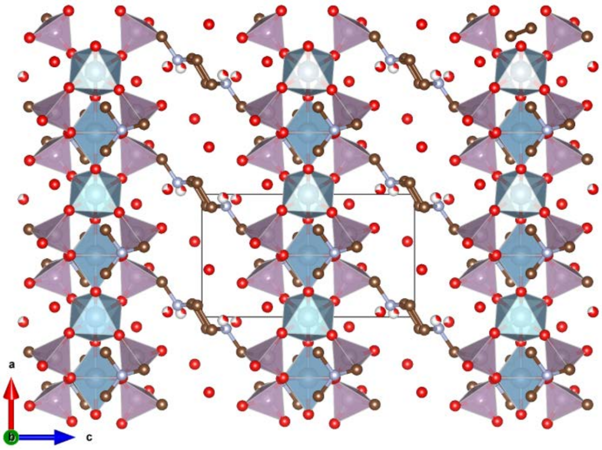 View parallel to *b*-axis

C2/m	*P-1*
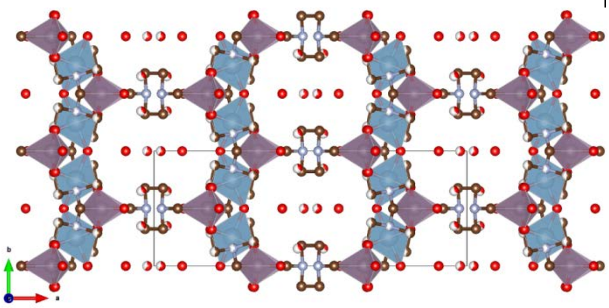	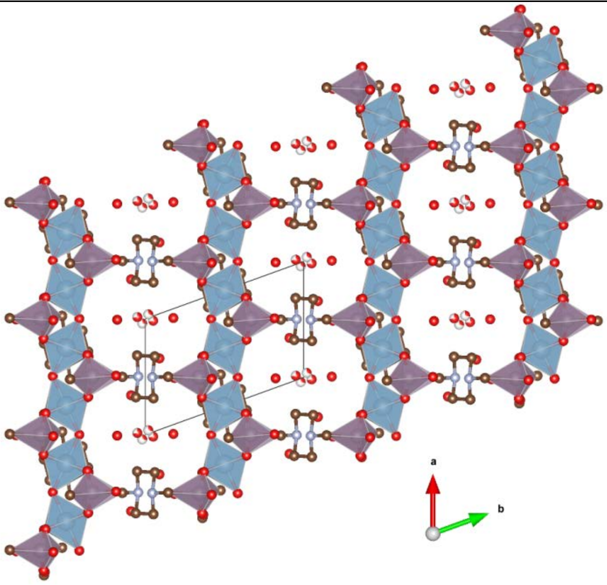
View parallel to *c*-axis<br>(N.B. *c*-axis out of page)	View parallel to *c*-axis<br>(N.B. *c*-axis in to page)

Fig. S8.1. Comparison of the crystal structures of MIL-91(Al) in the literature monoclinic *C2/m* cell[9] (left) and the new triclinic *P-1* cell reported in this work. Structurally there is very little difference between the two models, but crucially disorder has been removed in the new structure.

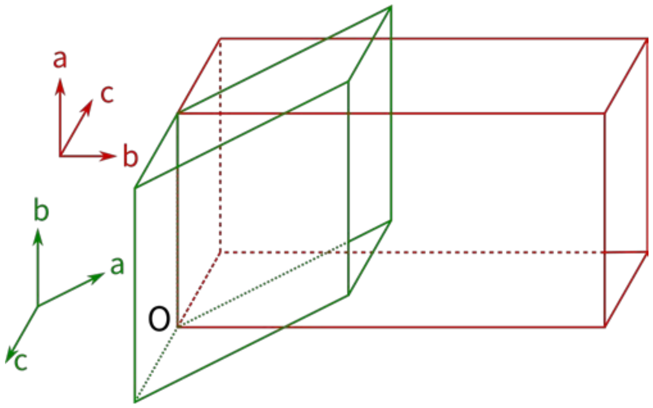

Fig. S8.2. Schematic of showing the two cells overlaid on top of one another. The monoclinic *C2/m* cell of Serre *et al.* is shown in red and the new triclinic *P-1* cell is shown in green. Note that to maintain the right-hand axis set, the orientation of the *c*-axis has changed by 180°.

S9 Porosity of MIL-91(Al) & Effect of Activation on the Structure

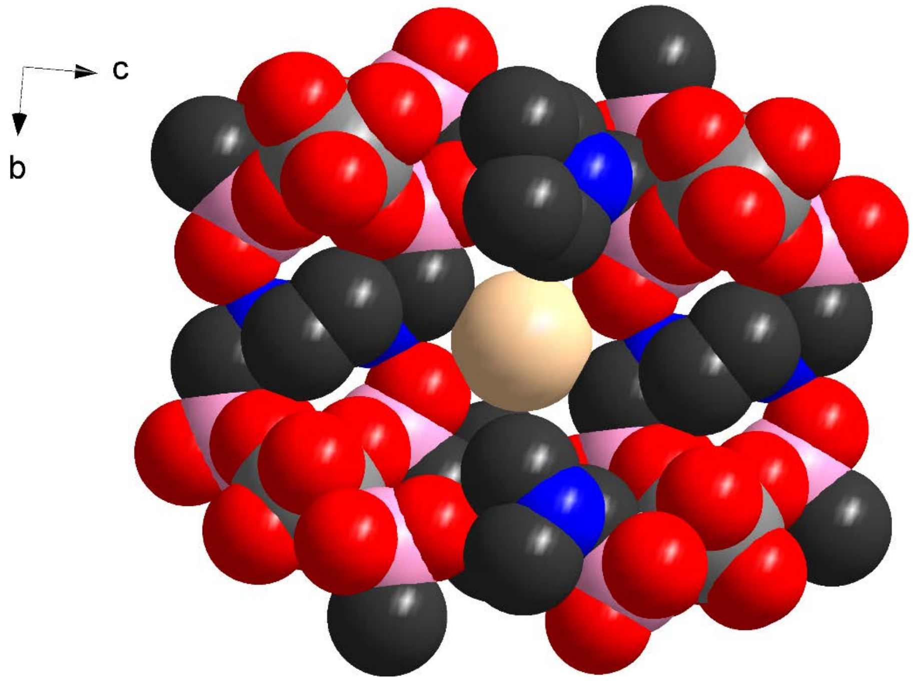

Figure S9.1. Space filling model of one channel of MIL-91(Al), view along the *a*-axis. A sphere of 3.5 Å in diameter is added to demonstrate the pore size. O in red, N in blue, C in black, Al in grey and P in pink.

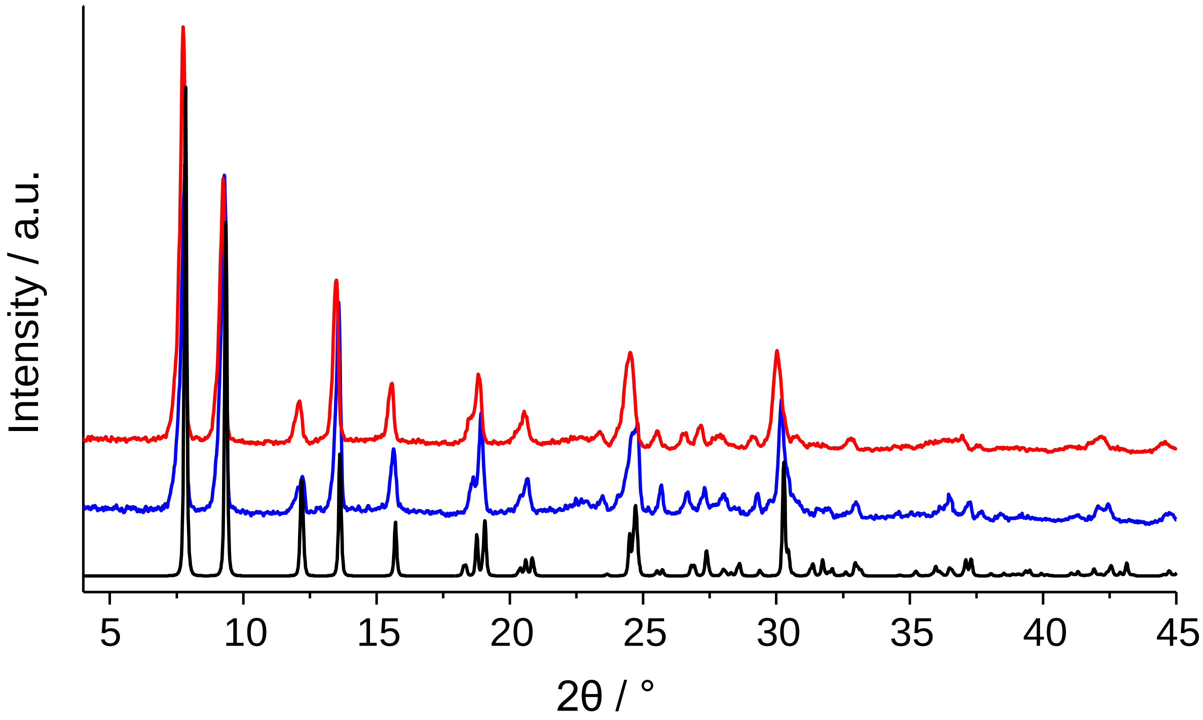

Figure S9.2 Comparison of the PXRD patterns of MIL-91(Al) as-synthesized (blue curve) and after the H_2O sorption measurements (red curve) with a simulated PXRD pattern (black curve).

S10 References

[1] D. Villemin, B. Moreau, A. Elbilali, M.-A. Didi, M. Kaid, P.-A. Jaffrès, *Phosphorus Sulfur Silicon Relat. Elem.* **2010**, *185*, 2511–2519.

[2] F. Rouquerol, J. Rouquerol, K. S. W. Sing, *Adsorption by Powders and Porous Solids: Principles, Methodology and Applications*, Academic Press, **1998**.

[3] J. Rouquerol, P. L. Llewellyn, F. Rouquerol, in *Charact. Porous Solids VII Proc. 7th Int. Symp. Charact. Porous Solids COPS-VII Aix-En-Provence Fr. 26-28 May 2005* (Eds.: P.L. Llewellyn, F. Rodriquez-Reinoso, J. Rouquerol, N.A. Seaton), Elsevier, **2007**, pp. 49–56.

[4] N. Stock, *Microporous Mesoporous Mater.* **2010**, *129*, 287–295.

[5] S. P. Thompson, J. E. Parker, J. Potter, T. P. Hill, A. Birt, T. M. Cobb, F. Yuan, C. C. Tang, *Rev. Sci. Instrum.* **2009**, *80*, 75107–9.

[6] A. Coelho, *TOPAS-Academic v5*, Coehlo Software, Brisbane, Australia, **2012**.

[7] P. Thompson, D. E. Cox, J. B. Hastings, *J. Appl. Crystallogr.* **1987**, *20*, 79–83.

[8] P. Stephens, *J. Appl. Crystallogr.* **1999**, *32*, 281–289.

[9] C. Serre, J. A. Groves, P. Lightfoot, A. M. Z. Slawin, P. A. Wright, N. Stock, T. Bein, M. Haouas, F. Taulelle, G. Férey, *Chem. Mater.* **2006**, *18*, 1451–1457.

[10] *Materials Studio v4.3*, Accelrys, San Diego, USA; Cambridge, UK; Tokyo Japan, **2008**.

[11] A. Spek, *J. Appl. Crystallogr.* **2003**, *36*, 7–13.

[12] R. W. Cheary, A. A. Coelho, *J. Appl. Crystallogr.* **1998**, *31*, 851–861.

[13] R. W. Cheary, A. A. Coelho, *J. Appl. Crystallogr.* **1998**, *31*, 862–868.
